# Unlocking the Path to Healthier Families: The Untapped Potential of Men’s Preconception Health

**DOI:** 10.1007/s10935-023-00762-y

**Published:** 2023-11-28

**Authors:** Muna Abed Alah

**Affiliations:** Community Medicine Department, Hamad Medical Corporation (HMC), P.O. Box 3050, Doha, Qatar

**Keywords:** Preconception, Men, Primary healthcare, Health promotion

## Abstract

This debate paper explores the necessity of introducing a comprehensive primary care model for men’s preconception health. It highlights the importance of a holistic approach that includes risk assessment, health promotion, and clinical and psychological interventions. Despite the current limited focus on male preconception health in primary care, there is evidence suggesting a growing awareness among men about the importance of optimizing their health before conception. The paper stresses the importance of such a model in addressing various aspects of men’s well-being, family dynamics, and overall reproductive health outcomes. It also acknowledges potential limitations and considerations related to implementing this crucial healthcare approach.

## Introduction

The concept of preconception health pertains to the health status of both males and females prior to pregnancy. Preconception care (PCC) aims to enhance the well-being of women and couples while mitigating biomedical, behavioral, and social factors that contribute to unfavorable maternal and child health outcomes through a set of evidence-based interventions (World Health Organization (WHO). While preconception health recommendations extend to all individuals of reproductive age, it is noteworthy that the bulk of interventions and messaging predominantly concentrate on women (Robbins et al., 2016; Thompson et al., 2017; Mello et al., 2020), Such an emphasis may inadvertently reinforce prevailing societal beliefs that women bear the primary responsibility for safeguarding children’s health (Mello et al., 2019, 2020; McGowan et al., 2020). In a study that explored the health behaviors, attitudes, and beliefs of adults of reproductive age in regard to preconception health in Irland, the predominant view among participants highlighted the significance of preconception care, with a stronger inclination towards the necessity for women, as opposed to men, to adopt health-promoting behaviors prior to conception (Cassinelli et al., 2023). Studies conducted with male participants in the United Kingdom, and the United States, have indicated a common perception among men that their opinions are often overlooked in matters of reproductive health, leading to a predominant focus on women in such discussions (Hamm et al., 2019; Grace et al., 2019). However, researchers are stressing upon the significance of extending preconception care to men as several studies have highlighted the influence of paternal health factors, including obesity, cardiovascular health, and work conditions, on birth outcomes (Davey Smith et al., 2007; Hillman et al., 2013; Cassinelli et al., 2023). A recently published systematic review showed that primary care-based preconception interventions are effective in improving health knowledge and reducing preconception risk factors in women, with limited evidence for their effectiveness in men indicating a need for further research in this area. (Withanage et al., 2022)

## The Far-Reaching Implications of Men’s Preconception Health

Improving men’s overall health can enhance their biological and genetic contributions to conception by improving sperm quality. Recent studies suggest that paternal preconception exposure to environmental and occupational factors, such as heat, radiation, and endocrine disruptors, can significantly impact sperm quality (Kotelchuck, 2022). This exposure is associated with adverse outcomes in offspring, including birth defects, malignancies, and developmental concerns (Kothari et al., 2019). Enhancing men’s preconception health positively influences pregnancy outcomes, with impacts extending beyond sperm quality to broader biological and genetic contributions (Kotelchuck & Lu, 2017). Recent studies, including animal models, have shown that men’s preconception stress can alter epigenetic patterns, affecting their offspring’s mental health (Mychasiuk et al., 2013; Kotelchuck & Lu, 2017). Furthermore, research showed how men’s preconception physical health conditions, like diabetes, can have developmental impacts on children. There was an observed decrease in gestational age for infants whose fathers had a medical history of diabetes and a higher frequency of fast food consumption (Moss & Harris, 2015). Paternal obesity is also a major risk factor for chronic diseases in children, as it can lead to preterm birth and fertility issues (Houfflyn et al., 2017). Studies have explored inter-generational and trans-generational epigenetic effects in sperm cells and offspring, with the paternal influence depending on body mass index and the transfer of pre-conceptional environmental influences through the sperm epigenome (Kothari et al., 2019). Therefore, optimizing weight in fathers before conception is critical. Furthermore, the behavioral practices of men, particularly their smoking habits, play a crucial role in pregnancy outcomes. When pregnant women are exposed to second-hand smoke due to their male partners’ smoking, it negatively affects fetal development and is associated with a range of poorer birth outcomes (Kotelchuck & Lu, 2017).

Comprehensive preconception care for men can facilitate family planning, enhance the reproductive health of their female partners, and prepare men for their roles as fathers (Frey et al., 2012). Men are inherently vital participants in family planning, and fostering their active and constructive involvement in decision-making processes is crucial. This is particularly significant as pregnancies that are planned and wanted tend to result in better birth outcomes and heightened parental dedication to the health and well-being of their child, spanning from prenatal to postnatal care (Kotelchuck & Lu, 2017). Providing preconception health care to men enables the identification and management of sexually transmitted infections (STIs), as well as exposure to certain environmental toxins. Addressing these issues not only improves the reproductive health of men but also has a positive impact on the reproductive biology of women and their fetuses in both current and future pregnancies. It is crucial to note that treating STIs in women is likely to be ineffective without the concurrent treatment of their male partners (Kotelchuck & Lu, 2017; Rabiei et al., 2022).

Most importantly, preconception care offers an opportunity, similar to the opportunity it presents for women, for disease prevention and health promotion (Frey et al., 2008). Just as with women, the pregnancy and early parenthood phase may present a prime opportunity to focus on men’s overall health needs. Sparse and singular self-reported evaluations of fathers’ health status during preconception and prenatal phases indicate significant potential for enhancing men’s physical health and their engagement with healthcare services (Kotelchuck, 2022). by taking responsibility for their health before conception, men are also taking a proactive role in the health and well-being of their family. This early engagement in family health is a step toward balancing the gender roles in family care, traditionally skewed towards women, and promotes a more equitable distribution of responsibility between men and women in family life and child-rearing (Kotelchuck, 2022). Moreover, it is becoming evident that preconception care for men holds the potential to improve reproductive health practices and outcomes for women as well. Men often influence decisions regarding prenatal care, delivery services, and other health-seeking behaviors, underscoring their pivotal role in the broader context of family health (Frey et al., 2008).

## Emphasizing the Need for a Men’s Preconception Care Model in Primary Care: Considerations and Limitations

A comprehensive approach to men’s preconception care encompasses risk assessment through comprehensive history-taking and physical examination, health promotion (including weight management, nutrition, stress reduction, resilience enhancement, and immunization), and both clinical and psychological interventions (Frey et al., 2008).This approach should include regular STI screening, especially for those at higher risk, and ensuring all vaccinations are current to prevent illnesses that could impact pregnancy outcomes (Nypaver et al., 2016). Lifestyle modifications are crucial, with support provided for smoking cessation, counseling on reducing alcohol and substance use, promoting a healthy diet and regular exercise for weight management, and offering strategies for stress management (Mitchell & Verbiest, 2013; Nypaver et al., 2016; Iftikhar Ahmad & Ahmad, 2019). Men should be educated about fertility, family planning options, and effective contraception methods for those not immediately planning a pregnancy (Mitchell & Verbiest, 2013). Chronic diseases such as diabetes, hypertension, and thyroid disorders should be well-managed, with a thorough review of medications that might affect fertility or pregnancy outcomes. Assessment of exposure to environmental and occupational toxins is vital, with guidance provided to minimize exposure to harmful substances (Nypaver et al., 2016). Regular follow-ups are necessary to monitor progress, adjust care plans, and provide referrals to specialists when needed. Additionally, providing educational resources, emotional support, and considering referral to mental health services if necessary are essential components of comprehensive preconception care for men.

Despite the limited attention given to male preconception health at primary healthcare settings in current literature, some evidence suggests that many men recognize the importance of optimizing their health before conception and express a preference for receiving preconception guidance from their family physician (Frey et al., 2012). In light of this, the establishment of a comprehensive primary health preconception model for men that takes into account sexual health, overall well-being, fathering, child development, relationship dynamics, and access to services becomes imperative. Such an approach is essential to revitalize the role of preconception care for men and to harness its benefits for male health and family health as a whole (O’Brien et al., 2018). I would propose a two-pronged strategy for incorporating preconception health into men’s primary healthcare framework. This strategy embeds preconception health assessments as a routine element of all primary care visits for men, which is crucial given the prevalence of unplanned pregnancies and men’s irregular engagement with healthcare services. Simultaneously, establishing specialized preconception care clinics that not only cater to individuals actively planning for pregnancy but also facilitate the co-presentation of couples, ensuring that both partners receive comprehensive preconception guidance together. This inclusive model ensures that preconception health becomes an integral part of healthcare for all men, supporting those who might become fathers unexpectedly and those who are intentionally preparing for fatherhood alongside their partners. To effectively implement the proposed two-pronged strategy for preconception care, it is essential to evaluate men’s intentions regarding pregnancy. Men with a desire to conceive in the near future should be referred to specialized clinics where they and their partners can receive a comprehensive suite of preconception services, designed to optimize the health of both prospective parents. Conversely, for those without immediate plans for pregnancy, primary healthcare visits will include a focused preconception counseling session that primarily addresses men’s health, ensuring they are informed and prepared for future reproductive decisions. To ensure the consistent delivery of preconception care within the proposed dual approach, primary care providers should be thoroughly trained and follow clear protocols to integrate preconception health inquiries into routine visits. Electronic Health Records (EHR) should be optimized with prompts to ensure preconception health is addressed at every opportunity. Patient education initiatives are crucial to cultivate an understanding and appreciation for preconception health among men. Additionally, comprehensive follow-up and referral systems must be in place to maintain continuity of care and guide men to appropriate preconception health services as needed.

## Challenges to Men’s Involvement in Preconception Care in Primary Healthcare Settings

Fostering awareness and action towards men’s preconception health within the framework of primary healthcare is a vital step toward enhancing overall reproductive and family health outcomes. However, incorporating a preconception primary healthcare model tailored for men may face several noteworthy challenges. These challenges encompass limited awareness and comprehension among men regarding the significance of preconception care, potentially resulting in reduced utilization of such services (Rabiei et al., 2023). Additionally, healthcare systems might not be suitably equipped or funded to provide specialized preconception care for men, given the traditional emphasis on women’s reproductive health. Constrained resources, the absence of national health insurance that covers screenings and diagnostic evaluations, and the unavailability of necessary counseling and treatments hinder providing the service (Zhou et al., 2016). Moreover, the high incidence of unplanned pregnancies in even affluent settings often leads to seeking preconception advice from primary care physicians only after pregnancy. Overcoming societal norms and addressing potential stigmas related to male involvement in preconception care presents another hurdle. Furthermore, ensuring healthcare providers are adequately trained and proficient in addressing the specific preconception health needs of men is imperative (Hogg et al., 2019). It is essential to note that these factors must be thoughtfully considered and addressed when developing and implementing men’s preconception primary healthcare model through a series of strategic solutions. Improving health literacy and educating men about the importance of preconception health is crucial, and this can be achieved through public health campaigns and accessible information that highlights the role of preconception care in fertility and overall family health (Whooten et al., 2023). While there is evidence of improved awareness of preconception health among men, particularly those of higher socioeconomic status and health consciousness, the journey towards widespread understanding and active engagement is still in its infancy. Studies indicating a rise in awareness represent a positive shift, yet they also emphasize the vast scope of work that remains. Most men, as noted, continue to be surprisingly uninformed about their significant contributions to child and maternal health outcomes (Rabiei et al., 2023). It is imperative that we extend these efforts beyond the more informed and health-conscious individuals to reach a broader demographic, ensuring that all men are equipped with the knowledge and resources necessary to contribute positively to preconception health. Reforming healthcare systems to provide preconception care for men and transiting from maternal-focused to couple-focused preconception care is also essential, which requires investment in services that have traditionally been focused on women’s reproductive health. Changing societal norms is another critical step; by actively working to dismantle stigmas and promote the concept of shared responsibility in reproductive health, men can feel more empowered to engage in preconception care. Lastly, ensuring that healthcare providers are well-trained to address men’s specific preconception health needs will help in providing comprehensive care. These concerted efforts can lead to a more inclusive approach to preconception care, ultimately benefiting men’s health and the health of their future children.

In summary, Preconception care for men can improve reproductive justice for women by promoting shared responsibility for reproductive health and outcomes. When men are informed and engaged in preconception health practices, it can lead to better family planning decisions, healthier pregnancies, and improved maternal health outcomes. This shared approach helps to alleviate the disproportionate burden often placed on women to manage contraception, pregnancy planning, and childbearing. By ensuring that men are equally informed and involved, preconception care for men supports a more equitable distribution of reproductive labor and decision-making, which is a core aspect of reproductive justice. This not only benefits women’s health and autonomy but also fosters a more supportive environment for both parents throughout the preconception and pregnancy journey.

I hope that the insights presented in this article prompt further exploration and commitment to this critical aspect of public health.

## Data Availability

Not applicable.
